# Organic Photovoltaics: More than Ever, an Interdisciplinary Field

**DOI:** 10.3390/polym8030070

**Published:** 2016-03-02

**Authors:** Laure Biniek, Christian B. Nielsen

**Affiliations:** 1 Institut Charles Sadron, CNRS-Université de Strasbourg, 23 rue du Loess, 67034 Strasbourg, France; 2 Department of Chemistry and Centre for Plastic Electronics, Imperial College London, London SW7 2AZ, UK

## 1. Introduction

Despite the growing interest and rapid advancement of alternative photovoltaic (PV) technologies such as perovskite based PV devices, we still believe that organic photovoltaic (OPV) devices have a significant potential for stable, low-cost solar power generation. As a matter of fact, Heliatek announced a new organic photovoltaic world record efficiency of 13.2% in the beginning of February 2016, highlighting the continued interest in OPV technology from both academia and industry [1]. The success was claimed to be based on the close cooperation of chemists and physicists which demonstrates the importance of the interdisciplinarity in this research field.

The Special Issue on organic photovoltaics that follows highlights the complexity of the issues facing researchers and engineers involved in OPV research and development. Interestingly, the author contributions cover the field from macromolecular engineering to device elaboration and characterization, not forgetting active layer morphological control, characterization of bulk heterojunction (BHJ) solar cells and bilayer device architectures.

In terms of molecular and macromolecular engineering, two interesting contributions discuss new methods to tune the optoelectronic properties of the electron donor polymer, while a third contribution is proposing an analysis of the effect of molecular architecture on photocurrent generation. First, Bronstein *et al*. present a novel approach based on the use of spirocyclic systems in the conjugated backbone of electron donor polymers to tune the energy levels of the materials [2]. Modifications of the C10-heteroatom on the anthracene unit affect the energy levels and, thus, absorption and emission properties of the polymers. Leclerc *et al*. review extensively the various and sometimes contradicting effects of backbone fluorination of conjugated polymers in use for solar cells [3]. The discussion covers synthesis, frontier molecular orbitals, active layer morphology and device performances. Finally, Dimitrov *et al*. point out the importance of singlet exciton lifetime in electron donor polymers in regards to device performances, *i.e*., photocurrent generation [4]. The authors propose an analysis on the dependence of singlet exciton lifetime on molecular properties, such as polymer band gap and crystallinity.

Regarding the active layer morphology, donor–acceptor phase separation control is still a key issue in the OPV area. To face this problem, Jang *et al*. propose to (re)consider bilayer architecture with an optimized sequential solution deposition technique, allowing the control of the morphology at the single interface between the electron donor polymer and the acceptor molecular system [5]. The process leads to an increase in the internal charge collection efficiency and might allow the reassessment of donor polymers which have shown insufficient performances in BHJ architectures.

Dyck *et al*. have also been working on the issue of the donor–acceptor phase separation in BHJ systems, but from a characterization point of view [6]. They demonstrate the application of two electron energy-loss spectroscopic imaging techniques to produce a carbon and sulfur density map of the active layer. Quantitative measurement of the fraction of each of the donor and acceptor materials can thus be obtained.

Finally, Owens *et al*. point out the importance of organic solar cells lifetime problems and the lack of outdoors tests and data [7]. The authors propose an interlaboratory approach to test different device architectures under real outdoor conditions for more than six months. They also discuss the degradation pathway of the devices in outdoor conditions, with a comparison of devices kept in a controlled atmosphere, and also in the dark.

To conclude, in order to reach both high efficiency and long-term stability of organic solar cells, only a well-balanced optimization of all the different criteria (materials properties, morphology and device architectures) will be effective. For this reason, it is important to encourage interdisciplinarity and interlaboratory collaboration approaches to face all the remaining issues preventing the pre-industrialization step of the OPV area.

## References

[1] Heliatek sets new Organic Photovoltaic world record efficiency of 13.2%. http://www.heliatek.com/en/press/press-releases.

[2] Bronstein H., King F.D. (2016). Energetic tuning in spirocyclic conjugated polymers. Polymers.

[3] Leclerc N., Chávez P., Ibraikulov O.A., Heiser T., Lévêque P. (2016). Impact of backbone fluorination on π-conjugated polymers in organic photovoltaic devices: A Review. Polymers.

[4] Dimitrov S.D., Schroeder B.C., Nielsen C.B., Bronstein H., Fei Z., McCulloch I., Heeney M., Durrant J.R. (2016). Singlet exciton lifetimes in conjugated polymer films for organic solar cells. Polymers.

[5] Jang Y., Seo J.-W., Seok J., Lee J.-Y., Kim K. (2015). Roughening conjugated polymer surface for enhancing the charge collection efficiency of sequentially deposited polymer/fullerene photovoltaics. Polymers.

[6] Dyck O., Hu S., Das S., Keum J., Xiao K., Khomami B., Duscher G. (2015). Quantitative phase fraction detection in organic photovoltaic materials through EELS imaging. Polymers.

[7] Owens C., Ferguson G.M., Hermenau M., Voroshazi E., Galagan Y., Zimmermann B., Rösch R., Angmo D., Teran-Escobar G., Uhrich C. (2016). Comparative indoor and outdoor degradation of organic photovoltaic cells via inter-laboratory collaboration. Polymers.

